# Association between disability and cognitive function in older Chinese people: a moderated mediation of social relationships and depressive symptoms

**DOI:** 10.3389/fpubh.2024.1354877

**Published:** 2024-04-16

**Authors:** Fangzhu Ai, Enguang Li, Aohua Dong, Huijun Zhang

**Affiliations:** Department of Nursing, Jinzhou Medical University, Jinzhou, China

**Keywords:** disability, cognitive impairment, depressive symptoms, social relationships, mediation, moderate

## Abstract

**Objective:**

Many previous studies have found that disability leads to cognitive impairment, and in order to better understand the underlying mechanisms between disability and cognitive impairment, the present study aimed to investigate the moderating role of social relationships, including their role as mediators between disability and cognitive impairment in depressive symptoms.

**Study design:**

This is a cross-sectional study.

**Methods:**

A total of 5,699 Chinese older adults from the 2018 China Longitudinal Healthy Longevity Survey (CLHLS) were included in this study, and PROCESS macro was used to perform simple mediator and moderator mediator analyses, which were used to analyze the relationship between depressive symptoms and social relationships between disability and cognitive impairment.

**Results:**

The results of this study showed significant correlations between disability, cognitive impairment, depressive symptoms, and social relationships, and that depressive symptoms mediated the relationship between disability and cognitive functioning [B = −0.232; 95% CI: (−0.304, −0.164)], and that social relationships mediated disability and cognitive functioning through pathway a (Disability-Depressive Symptoms) [B = 0.190; 95% CI: (0.020, 0.036)], path b (depressive symptoms-cognitive impairment) [B = 0.029; 95% CI: (0.015, 0.042)], and path c’ (incapacitation-cognitive impairment) [B = 0.492; 95% CI: (0.298, 0.685)] to modulate the effect of incapacitation on cognitive impairment. In addition, social activities and social networks moderated the mediation model directly or indirectly, whereas social support moderated only the direct effect.

**Conclusion:**

This study explains the intrinsic link between incapacitation and cognitive impairment in Chinese older adults, and that social relationships and depressive symptoms can directly or indirectly modulate the effects between them. This provides a basis for healthcare professionals to be able to better develop interventions that can be used to improve the level of cognitive functioning and mental health of older adults.

## Introduction

Our country is faced with a severe situation of population aging. According to the latest national census data, the proportion of older adults in China has reached 14 percent, making it one of the countries with the largest older adults in the world (1). According to the global population data, by 2050, the global older adults will reach 2 billion, and society will usher in a stage of deep aging. At that time, the proportion of China’s older adults will rise to 34.1% (2). In addition, with the aging of the population, the disability of older adults in China has also attracted wide attention. According to the sample survey data in 2016 (3), there were about 40.63 million disabled and semi-disabled older adults in China, accounting for 18.3% of the total older adults. Studies show that if nothing is done, this proportion will increase to 70% of the older adults by 2050 (4). Older adults face a higher risk of cognitive impairment due to limited activity and reduced physical function. Dementia will further increase the degree of disability in older adults. In recent years, a growing number of studies have shown that disability is an essential predictor of cognitive impairment (5, 6). According to a report by the World Health Organization, the number of people with cognitive impairment will double every 20 years, with most of them concentrated in developing countries (7). According to statistics, there are more than 50 million people with cognitive impairment in China, and the prevalence of mild cognitive impairment in older adults in China is 20.8%, which is four times the prevalence of dementia. Every year, 10–15% of patients with cognitive impairment will gradually progress to dementia, which will affect their daily lives to varying degrees and may even be life-threatening in severe cases (8–10). Cognitive impairment refers to the impairment of one or more functions in sensory, learning and memory, speech, attention, and executive function, and is a common symptom of nervous system diseases. Any factor that causes functional and structural abnormalities in the cerebral cortex can lead to cognitive impairment (11). In addition, studies have shown that by analyzing the development trend of population aging in the past 70 years since the founding of the People’s Republic of China, we can see that the aging trend in older adults in China has increased significantly (12). With the increase in life expectancy, the risk of cognitive impairment in older adults will also increase (13). It has been reported that early identification and intervention can effectively delay the deterioration of cognitive function, thereby significantly improving patients’ quality of life and life expectancy (14, 15). In a cohort study conducted in Chile, frailty in older adults was strongly associated with the development of cognitive impairment, and frailty was associated with a higher risk of death. Underlying cognitive impairment is a critical factor in the increased risk of death (16).

The incidence of depression is high in older adults. According to the statistics of the World Health Organization (17), the proportion of patients with depression in the total older adults is about 7–10%. In foreign countries, the prevalence of depression in older adults over 65 years old is usually 5–15%, while in China, the prevalence of depression in older adults is 6–33.5%. Especially when older adults have physical diseases, the incidence of depression can even be as high as 50% (18). Depression not only affects the quality of life of millions of older people but also imposes a long-term medical and care burden on families and society, causing significant stress (19). The proportion of depressive symptoms in the disabled older adults is relatively high in geriatric depression, which may be due to the limitation of daily activities, poor independent ability, and heavy disease burden. However, under the traditional family care model in China, the psychological status of the disabled older adults at home is often not paid enough attention, which may affect their health status and increase their degree of disability (20–22). In addition, Torres et al. conducted a 15-year follow-up study of 1,014 older adults aged 60 years and older. They showed that older adults with depressive symptoms were more likely to be at risk of disability (23). Some studies have suggested that there may be a bidirectional relationship between depression and disability (24). In addition, some studies have confirmed that depression is common in patients with cognitive impairment, and several studies have demonstrated the correlation between depression and cognitive frailty (25–27). Second, there are important studies demonstrating that frailty and the presence of many comorbidities in older adults are also associated with depression and cognitive impairment (28, 29).

Social relations, like defenders, provide help and support for individuals at different stages of life, escort life, enable individuals to successfully overcome difficulties and pressures, and promote personal physical and mental health (30). Good social relations can also promote the generation of positive emotions and improve the quality of life and happiness of life in older adults (31). Studies have shown that social relationships affect the cognitive function of older adults through different pathways. Still, the effects on the cognitive function of older adults may differ under different social and cultural backgrounds (32). Richer social relationships are associated with milder depressive symptoms and better cognitive function in older adults. Kelly et al. (32) summarized the effects of social activities, social networks, and social support on the cognitive function of older adults and defined the three as social relations. Social network refers to the structure of social relationships maintained by individuals, including close relationships with family and friends and formal relationships with other individuals and groups (33). Many previous studies have found that the more extensive the social network of older adults, the better their cognitive function (34, 35). Chinese researcher Li Feng et al. also found that older adults who lived alone, did not frequently communicate with friends and neighbors and lacked trusted friends had a significantly higher risk of dementia than other older adults (36). However, some studies have pointed out that despite the association between social networks and cognitive impairment, after a 10-year longitudinal survey, older adults reported that social networks did not alleviate cognitive impairment (37). Even the cross-sectional study by Krueger et al. (38) found no evidence that social networks were associated with cognitive conditions. In addition, social activities are also a form of social participation, which refers to the degree to which individuals participate in a wide range of social roles and relationships (39). Regular participation in cognitive and physical activities can improve cognitive function in older adults (40, 41). In addition, some studies have shown that social activities have a protective effect on cognitive function and cognitive impairment (42), but the relevant intervention studies are insufficient. Social support was divided into emotional support and instrumental support (43). Emotional support included “who to chat with at ordinary times” and “who to share with when you are worried. “A decrease in instrumental support measures, including “who to go to when you are in trouble, ““who will take care of you when you are ill, “and “whether children give you cash, “was found to predict cognitive decline 1 year later, mainly in speech, working memory, and executive function (44). We also found that previous studies have explored the relationship between frailty and cognitive function in Chinese older adults and the mediating role of depression and social relationships in the relationship between frailty and cognitive function (45). Frailty usually refers to physical weakness, inadequacy, and mental exhaustion. Disability has also been shown to be a significant predictor of cognitive impairment (5, 6). Disability refers to a physical or intellectual deficit, impairment, or disorder that limits or impairs the ability of an individual to perform major activities of daily living. In addition to their different definitions, the evaluation methods are also different. In contrast, disability is a more serious physical problem and may have a greater impact on cognitive function in older adults. Therefore, this study will further investigate the relationship between disability and cognitive function, which is of great practical significance.

**Figure 1:**
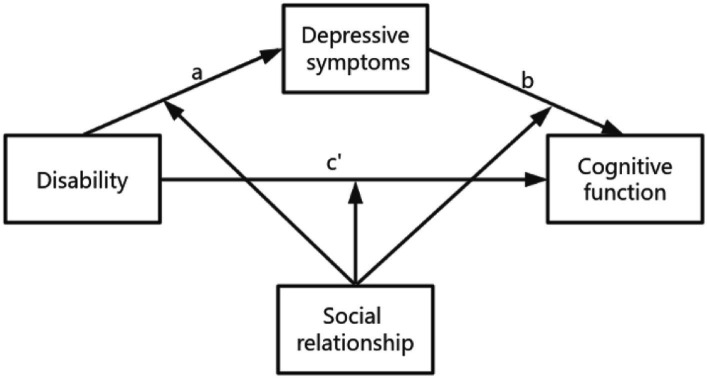
Moderation of the social relationship model as a mediating model of depression between disability and cognitive function (Andrew Hayes’ moderate-mediation model 59).

Therefore, the aims of this study were first to investigate whether depressive symptoms mediate the relationship between disability and cognitive impairment in older adults and, in addition, to examine whether social relationships (including the components of social relationships) directly or indirectly mediate the effect of disability on cognitive impairment. Based on the literature review, the following hypotheses and hypothesis models were proposed, as shown in Figure 1:

*Hypothesis 1*: Depressive symptoms mediate the association between disability and cognitive impairment.

*Hypothesis 2*: The direct and indirect associations between disability and cognitive impairment are moderated by social relationships and mediated by depressive symptoms.

*Hypothesis 3*: The direct and indirect associations between disability and cognitive impairment will be moderated by social activities, social networks, and social support, with depressive symptoms acting as a mediator.

## Materials and methods

### Participants

**Figure 2:**
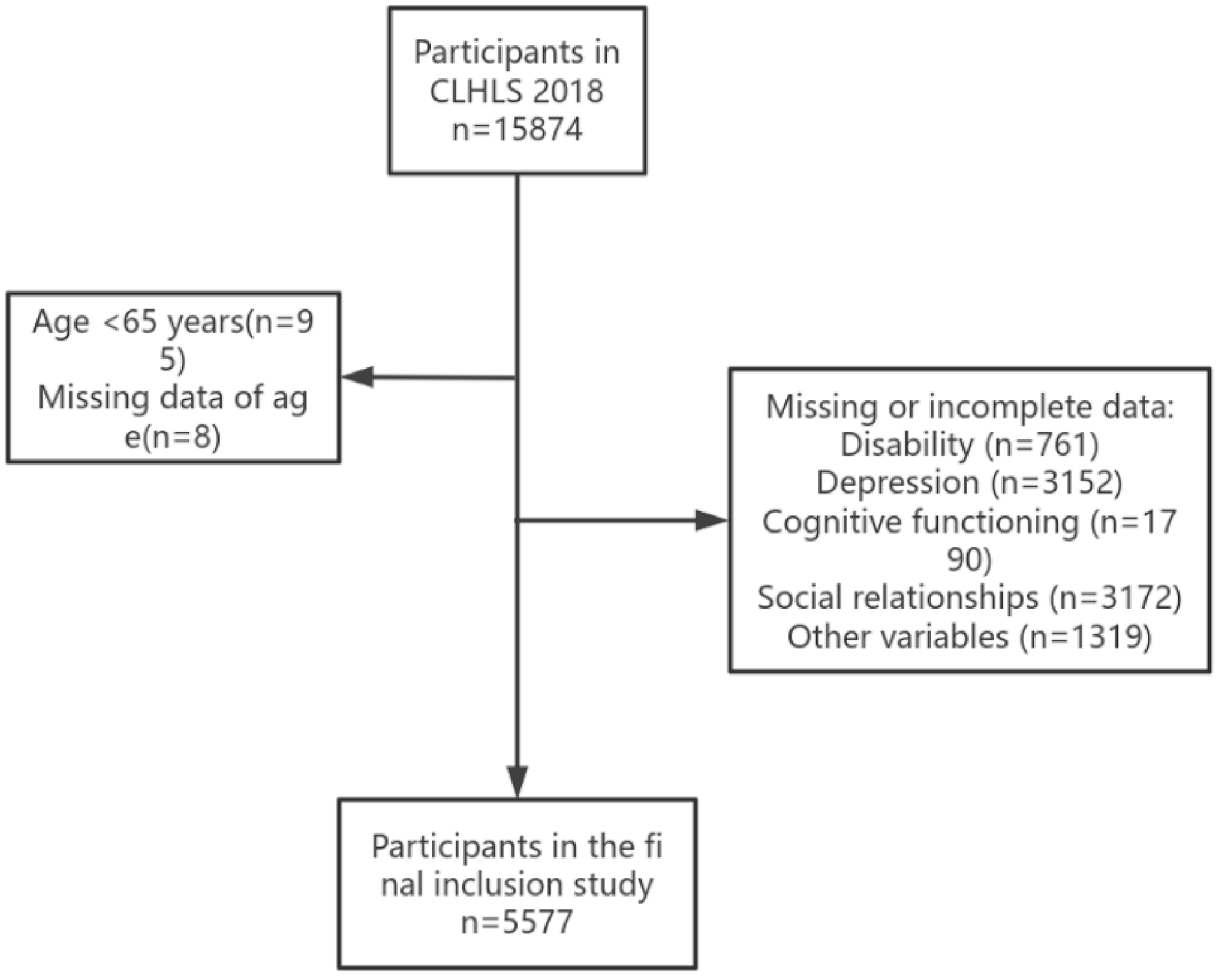
Flow chart of the selection of study participants.

The data used in this study were from the Chinese Longitudinal Healthy Longevity Survey (CLHLS) in 2018. The program began in 1998 and was followed up every 3–4 years. The survey covered 23 provinces in China, and older adults aged 65 years and above were surveyed by face-to-face interviews. Eight surveys have been conducted since 1998, with the most recent follow-up completed in 2017–2018. All respondents who participated in the survey volunteered and signed informed consent during the survey. For older adults who could not sign, the family members signed the informed consent form (46). The study was approved by the Biomedical Ethics Committee of Peking University (IRB00001052-13074). In addition, our team has applied for and obtained the right to use the data through the Open Research Data Platform of Peking University. To reflect the current situation of older people, we used data from the 2018 CLHLS project involving 15,874 participants. During data screening, we excluded participants who did not complete the Mini-Mental State Examination (MMSE) and those with missing data on depression, disability, or social relationships. At the same time, we excluded some participants due to missing data on relevant covariates and finally determined 5577older adults as the sample size. The specific screening process is shown in Figure 2.

### Dependent variables

#### Cognitive function

The Mini-Mental State Examination (MMSE) was used to measure cognitive function in CLHLS (47). The scale consists of 24 questions covering five domains: general ability, responsiveness, attention and calculation, recall, language comprehension, and self-coordination. Participants received 1 point if they answered correctly or 0 points if they did not. Among them, the sixth question is “Name the amount of food in 1 min,” and the highest score is 7 points. Total scores range from 0 to 30, with higher scores indicating better cognitive function in older adults. According to previous studies, participants with MMSE scores less than 24 were defined as having cognitive impairment (48, 49). The MMSE has been widely used in many studies and has shown good reliability (50). In this study, the Cronbach’s α coefficient of the MMSE was 0.91, for more details on the Chinese version of the MMSE, please refer to Supplementary Table S1.

### Independent variables

#### Definition of disability

Disability refers to the limitation or loss of an individual’s ability to perform significant activities in daily life (51). The activities of daily living (ADL) (52) ability assessment scale was used to determine whether older adults were disabled. ADL disability was defined as needing assistance in one or more of the five essential activities (dressing, bathing, transferring, eating, toileting) or incontinence. Many previous studies have applied the scale to assess the activities of daily living in older adults (53, 54), and the validity and reliability of the scale are higher than 0.90 (53).

### Mediators

#### Assessment of depressive symptoms

CLHLS uses the CES-D10 scale to assess depressive symptoms in older adults, which was revised by Andresen et al. (55) and showed good reliability and validity in the Chinese older adults, with Cronbach’s α coefficient between 0.78 and 0.81. The scale consisted of 10 items and was divided into five grades. Negative items were assigned a value according to “always =3, often =2, sometimes =1, rarely or never =0,” while positive items were given the reverse value. Please refer to the following table for specific assignments. The higher the total score, the higher the degree of depression. When the total score ≥ 10, the presence of depressive symptoms was judged (55).

### Moderator

#### Assessment of social relations

According to previous studies, the score of social relations ranged from 0 to 13, which was divided into three parts: social activities (0 to 3), social networks (0 to 4) and social support (0 to 6, 32, 45, 56). The specific variables were assigned in Supplementary Table S2.

The definition of social ties was proposed by Berkman et al. (56) because many terms are used and interchanged more loosely when researchers discuss the impact of social ties on health. Berkman et al. proposed a unified definition to explain social relationships to clarify concepts and provide a consistent framework. According to their definition, social relationships include social networks, social activities, and social support (56). Social networks are defined as the network of social relationships surrounding an individual and the characteristics of those connections (56). Therefore, in this study, older adults-centered network was mainly considered, including four contents: the marital status of older adults, the living arrangement, the presence or absence of sibling visits, and the presence or absence of children visits. Social networks were scored on a scale of 0–4. Social support is usually divided into the following sub-types: emotional support, instrumental support, evaluative support, and informational support (57). Emotional support involves “love or care, compassion or understanding, and respect or value received from others.” (58) It is mainly provided by close people close to you, but less close people can also offer this support under limited conditions. In the theoretical framework proposed by Berkman (56), instrumental support refers to help with tangible needs, such as shopping, keeping appointments, making phone calls, cooking, cleaning, or paying bills. In contrast, House (59) defines instrumental support as help in kind, money, or labor. Evaluation support is associated with deciding which course of action to take. Informational support involves support that provides advice or information for specific needs. Therefore, in this study, six aspects of social support were included: whether older adults had someone to talk to, someone to share ideas with, someone to ask for help, someone to care for when sick, and financial support from sons and daughters. Scores ranged from 0 to 6. Social activities, also known as social participation, include meeting with friends, attending social events, taking on social roles, participating in group recreational activities, and attending religious services. In this study, social participation covered the following three dimensions: participation in playing cards or mahjong, participation in organized activities, and experiences of participation in various types of activities. Scores range from 0 to 3.

### Covariates

Covariates included sociodemographic, psychological, behavioral, and disease characteristics. Socio-demographic characteristics include age (years), gender (0 = female 1 = male), marriage (0 = unmarried 1 = married), education (0 = no education 1 = educated), exercise (0 = no exercise 1 = exercise), place of residence (1 = city 2 = town or rural), and financial support (0 = not enough 1 = enough). Psychological characteristics include anxiety, and the GAD-7 scale was applied in the CLHLS to assess anxiety in older adults. The GAD-7 scale has been validated for use in Chinese populations (60), and the scale has seven entries divided into four levels, all negative, according to the following scale: “none = 0, a few days = 1, more than half the time = 2, almost every day = 3,” with total scores assigned to “no = 0, some days = 1, more than half the time = 2, and almost every day = 3.” “The higher the total score, the more severe the anxiety symptoms. Behavioral characteristics included exercise (0 = No 1 = Yes), length of sleep (hours) and frailty. Frailty was defined by studying the Osteoporotic Fracture (SOF) index (61, 62), which consists of 3 components in the CLHLS (63), including (1) underweight (body mass index <18.5 kg/m?); (2) muscle strength (standing up from a chair without the aid of an arm); (3) low energy (to “Have you limited your activities in the past 6 months because of health problems?”). The disease data included whether older adults had other comorbidities, and 24 diseases were included in the clhls.

### Statistical analysis

In this study, the mean and standard deviation were used to describe the continuous variables, and the proportion and frequency were used to represent the categorical variables. For the difference test of continuous variables, the t-test was used. The chi-square test was used to test the difference between categorical variables. Correlations between disability, depressive symptoms, social relationships, and cognitive impairment were then analyzed. Finally, PROCESS macro (64) was used to analyze the mediation and moderated mediation models, including model 4 (to analyze the mediation), model 59 (to analyze the direct or indirect path in the mediation mediated by one variable), and model 76 (to analyze the direct or indirect path in the mediation mediated by two variables). These models are all based on the framework constructed by regression (65).

Firstly, Model 4 was used to examine whether depression mediates the relationship between disability and cognitive function. In addition, some covariates such as age, sex, marriage, residence, education, exercise, money, sleep duration, frailty, comorbidity, and anxiety were included in the mediation model. The mediation model was considered valid when the 95%CI of the indirect effect (a*b) did not include zero. Next, Model 59 was used to analyze whether social relationships played a moderating role in mediation. Mediation was divided into direct pathway (c’: disability—cognitive function) and indirect pathway (a: disability—depression, b: depression—cognitive impairment). Finally, Model 76 was used to analyze whether social networks, social activities, and social support played a moderating role in mediation. Except for marriage, financial support, and sleep duration for comorbidities, other variables were included in the mediating adjustment model. All analyses were performed in SPSS 27, and a *p*-value of less than 0.05 was used as the standard level of significance (two-sided tests).

## Results

### Characteristics of the participants

Based on the results in Table 1, 5,577 participants were included in this study, 2,588 (46.4%) men and 2,989 (53.6%) women. Among the participants with cognitive impairment, 288 (27.9%) were males and 746 (72.1%) were females. Therefore, it can be seen that older women are more likely to have cognitive impairment. In addition, living in urban or rural areas, being uneducated, having disability, depression, frailty, having other comorbidities, less social activities, and lack of exercise (*p* < 0.05) were more likely to have cognitive impairment. In addition, older age, longer sleep duration, and more severe anxiety and depression symptoms (*p* < 0.05) were also more likely to have cognitive impairment.

**Table 1:** Characteristics of study participants stratified by cognitive status.

Characteristic	Total (*n* = 5,577)	Normal cognition (*n* = 4,543)	Cognitive impairment (*n* = 1,034)	χ^2^ or t statistics	*p*
*Gender*				175.657	0.000
male	2,588 (46.4)	2,300 (50.6)	288 (27.9)		
female	2,989 (53.6)	2,243 (49.4)	746 (72.1)		
*Marriage*				365.619	0.000
unmarried	2,727 (48.9)	1944 (42.8)	783 (75.7)		
married	2,850 (51.1)	2,599 (57.2)	251 (24.3)		
*Place of residence*				35.619	0.000
city	1,265 (22.7)	1,103 (24.3)	162 (15.7)		
town or rural	4,312 (77.3)	3,440 (75.7)	872 (84.3)		
*Education*				651.994	0.000
Uneducated	2,347 (42.1)	1,546 (34)	801 (77.5)		
educated	3,230 (57.9)	2,997 (66)	233 (22.5)		
*Exercise*				112.133	0.000
no exercise	3,131 (56.1)	2,398 (52.8)	733 (70.9)		
exercise	2,446 (43.9)	2,145 (47.2)	301 (29.1)		
*Financial support*				14.847	0.000
not enough	696 (12.5)	530 (11.7)	166 (16.1)		
enough	4,881 (87.5)	4,013 (88.3)	868 (83.9)		
*Disability*				474.044	0.000
disabled	824 (14.8)	447 (9.8)	377 (36.5)		
non-disabled	4,753 (85.2)	4,096 (90.2)	657 (63.5)		
*Depressive symptoms*				96.832	0.000
non-depressed	4,216 (75.6)	3,557 (78.3)	659 (15.6)		
depressed	1,616 (24.4)	986 (21.7)	375 (36.3)		
*Frailty*					
no	3,076 (55.2)	2,791 (61.4)	285 (27.6)	390.709	0.000
yes	2,501 (44.8)	1752 (38.6)	749 (72.4)		
*Comorbidity*					
no	1,481 (26.6)	1,169 (25.7)	312 (30.2)	8.522	0.004
yes	4,096 (73.4)	3,374 (74.3)	722 (69.8)		
*Age*	82.12 ± 10.859	80.01 ± 10.027	91.39 ± 9.427	−33.295	0.000
*Length of sleep*	7.35 ± 2.211	7.3 ± 2.094	7.54 ± 2.66	−3.117	0.002
*GAD-7 score*	1.34 ± 2.627	1.24 ± 2.487	1.81 ± 3.128	−6.369	0.000
*CES-D-10 score*	7.02 ± 4.335	6.68 ± 4.17	8.55 ± 4.702	−12.734	0.000
*MMSE score*	26.36 ± 4.863	28.29 ± 1.799	17.92 ± 5.081	110.436	0.000
*Social relationships (score)*	8.16 ± 1.671	8.35 ± 1.645	7.33 ± 1.528	18.112	0.000
*Social activities*	0.5 ± 0.751	0.58 ± 0.787	0.17 ± 0.430	16.182	0.000
*Social networks*	2.46 ± 1.055	2.57 ± 1.045	1.97 ± 0.960	16.912	0.000
*Social support*	5.2 ± 0.929	5.20 ± 0.916	5.20 ± 0.982	0.148	0.883

### Correlation of disability, depressive symptoms, social relationships, and cognitive functioning

According to the results in Table 2, there were significant associations between disability, depressive symptoms, social relationships, and cognitive function (*p* < 0.05), but the relational component social support was not significantly associated with disability, cognitive function, social activities, and social networks (*p* < 0.05).

**Table 2:** Correlations for the main variables.

Variable	1	2	3	4	5	6	7
1. Disability	–						
2. Cognitive function	0.341**	–					
3. Depressive Symptoms	−0.103**	−0.197**	–				
4. Social activity	0.139**	0.247**	−0.166**	–			
5. Social networks	0.138**	0.258**	−0.148**	0.159**	–		
6. Social support	0.004	0.010	0.035**	−0.020	0.014	–	
7. Social Relationships	0.152**	0.279**	−0.149**	0.538**	0.711**	0.556**	–

*p < 0.05; **p < 0.01; ***p < 0.001.

### The relationship between disability and cognitive functioning

According to the results in Table 3, Model 1 analyzed the effect of disability on cognitive function, and the results showed that disability was negatively correlated with cognitive function (B = -4.695, *p* = 0.000 < 0.05). In model 2, we incorporated age, sex, marital status, place of residence, education level, exercise, financial support, sleep duration, frailty, comorbidity, and anxiety level to examine the effects of disability on cognitive function. The results showed that disability (B = −3.459, *p* = 0.000), age (B = −0.130, *p* = 0.000), place of residence (B = −0.440, *p* = 0.002), frailty (B = −0.841, *p* = 0.000), and anxiety (B = -0.109, *p* = 0.000) were negatively correlated with cognitive function, gender (B = 0.537, *p* = 0.000), education (B = 1.735, *p* = 0.000), exercise (B = 0.481, *p* = 0.000) and financial support (B = 0.354, *p* = 0.032) were positively correlated with cognitive function. Financial support, marital status, comorbidity and sleep duration were not significantly associated with cognitive function (*p* < 0.05).

**Table 3:** The association between disability and cognitive function.

	Model 1					Model 2				
	B	SE	p	LLCI	ULCI	B	SE	p	LLCI	ULCI
Disability	−4.695	0.171	0.000	−5.031	−4.359	−3.459	0.395	0.000	−4.233	−2.686
Age	–	–	–	–	–	−0.130	0.007	0.000	−0.143	−0.117
Gender	–	–	–	–	–	0.537	0.007	0.000	0.299	0.775
Marriage	–	–	–	–	–	0.079	0.165	0.635	−0.245	0.402
Place of residence	–	–	–	–	–	−0.440	0.141	0.002	−0.716	−0.164
Education	–	–	–	–	–	1.735	0.131	0.000	1.479	1.991
Exercise	–	–	–	–	–	0.481	0.115	0.000	0.255	0.707
Financial support	–	–	–	–	–	0.311	0.166	0.061	−0.015	0.637
Length of sleep	–	–	–	–	–	0.005	0.025	0.846	−0.044	0.053
Anxiety	–	–	–	–	–	−0.109	0.021	0.000	−0.151	−0.067
Frailty						−0.841	0.120	0.000	−1.076	−0.606
Comorbidity						0.161	0.122	0.187	−0.079	0.401

### Mediating effect of depressive symptoms between disability on cognitive functioning

Based on the results in Table 4, we used Model 4 to analyze the mediating effect of depressive symptoms on the relationship between disability and cognitive function. The results showed that the 95%CI of all paths did not include 0, indicating that these paths were significant. Specifically, disability and depression were positively correlated [B = 1.272, 95%CI = (0.953–1.591)] and negatively associated with depression and cognitive function [B = −0.186, 95%CI = (−0.213–−0.158)]. Disability was negatively correlated with cognitive function [B = −4.444, 95%CI = (−4.780–−4.109)]. In addition, a significant indirect effect of depression on the relationship between disability and cognitive function was found [B = −0.236, 95%CI = (−0.308–−0.165)].

**Table 4:** Testing the mediating effect of disability on cognitive function.

	Path	Model 4			
		B	SE	LLCI	ULCI
Total effect	Disability—Cognitive Function	−4.680	0.173	−5.018	−4.342
Direct effect	Disability—Depressive Symptoms	1.272	0.163	0.953	1.591
	Depressive Symptoms—Cognitive Function	−0.186	0.014	−0.213	−0.158
	Disability—Cognitive function	−4.444	0.171	−4.780	−4.109
Indirect effect	Disability—Depressive Symptoms—Cognitive function	−0.236	0.036	−0.308	−0.165

### Moderating mediating effects of disability on cognitive function

**Figure 3:**
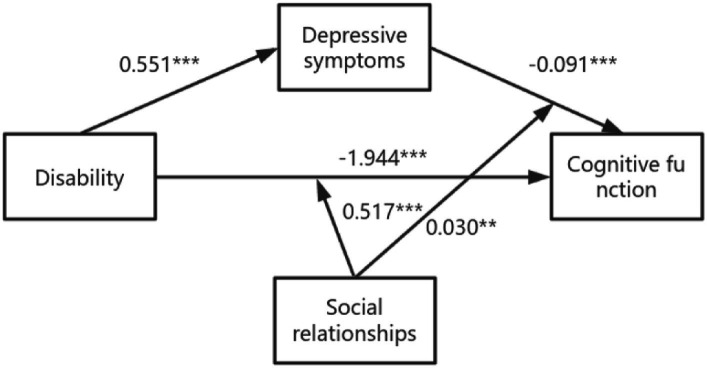
The final moderated mediation model: social relationships as moderator of the mediation model of depression between disability and cognitive function (Andrew Hayes’s mediation-moderation Model 15, *p < 0.05; **p < 0.01; ***p < 0.001). The moderated mediation model was controlled for covariates (age, gender, education, place of residence, exercise, financial support).

According to the results in Supplementary Table S3, Model 59 was used to analyze the moderating mediation model of disability on cognitive function. The results showed that the interaction between disability and social relations was positively correlated with cognitive function (B = 0.517, *p* = 0.000). It was also positively correlated with the interaction between depression and social relations (B = 0.030, *p* = 0.000). Thus, in paths a, b, and c ‘of this model, social relationships mediate the direct and indirect effects of disability on cognitive function. For details, please refer to Figure 3.

**Figure 4:**
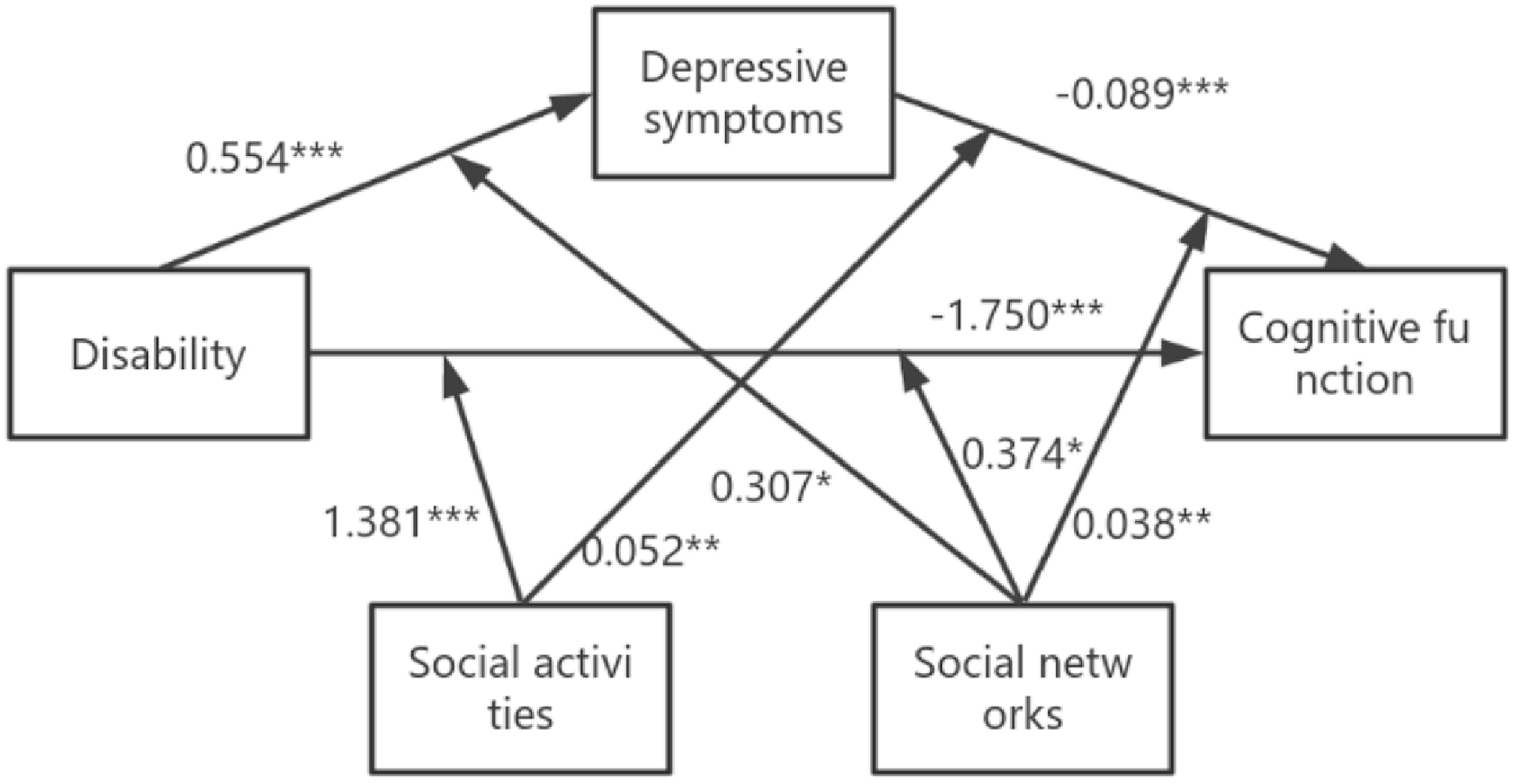
The final moderated mediation model: social activities and social networks as moderators of the mediation model of depression between disability and cognitive function (Andrew Hayes’s mediation-moderation Model 76, *p < 0.05; **p < 0.01; ***p < 0.001). The moderated mediation model was controlled for covariates (age, gender, education, place of residence, exercise, financial support).

According to the results in Supplementary Table S4, Model 76 was used to analyze the moderating effects of social activities and social networks on mediation. Results showed that social activities mediated the impact of disability on cognitive function through path b (social activities * disability, B = 1.381, *p* = 0.000) and path c’ (social activities * depression, B = 0.052, *p* = 0.003). Social network mediated the effect of disability on cognitive function through path a (social network * disability, B = 0.307, *p* = 0.038), path b (social network * depression, B = 0.038, *p* = 0.001), and path c’ (social network * depression, B = 0.374, *p* = 0.026). The specific model is shown in Figure 4. In addition, the moderating effect of social support on cognitive function was analyzed according to the results of Model59, and the results are shown in Supplementary Table S5. Social support only moderated the direct path (disability—cognitive function).

**Figure 5:**
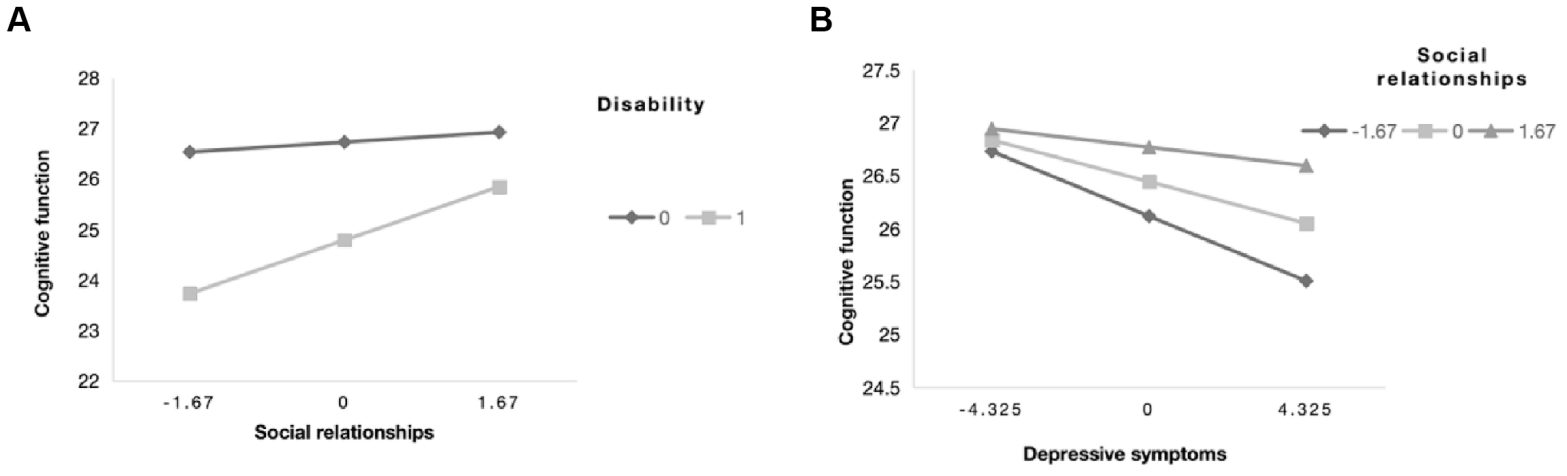
The simple plot of path **(A,B)** indicating the relationship between disability (0 = non-disabled,1 = disabled), depressive symptoms, and cognitive impairment among different levels of social relationships.

**Figure 6:**
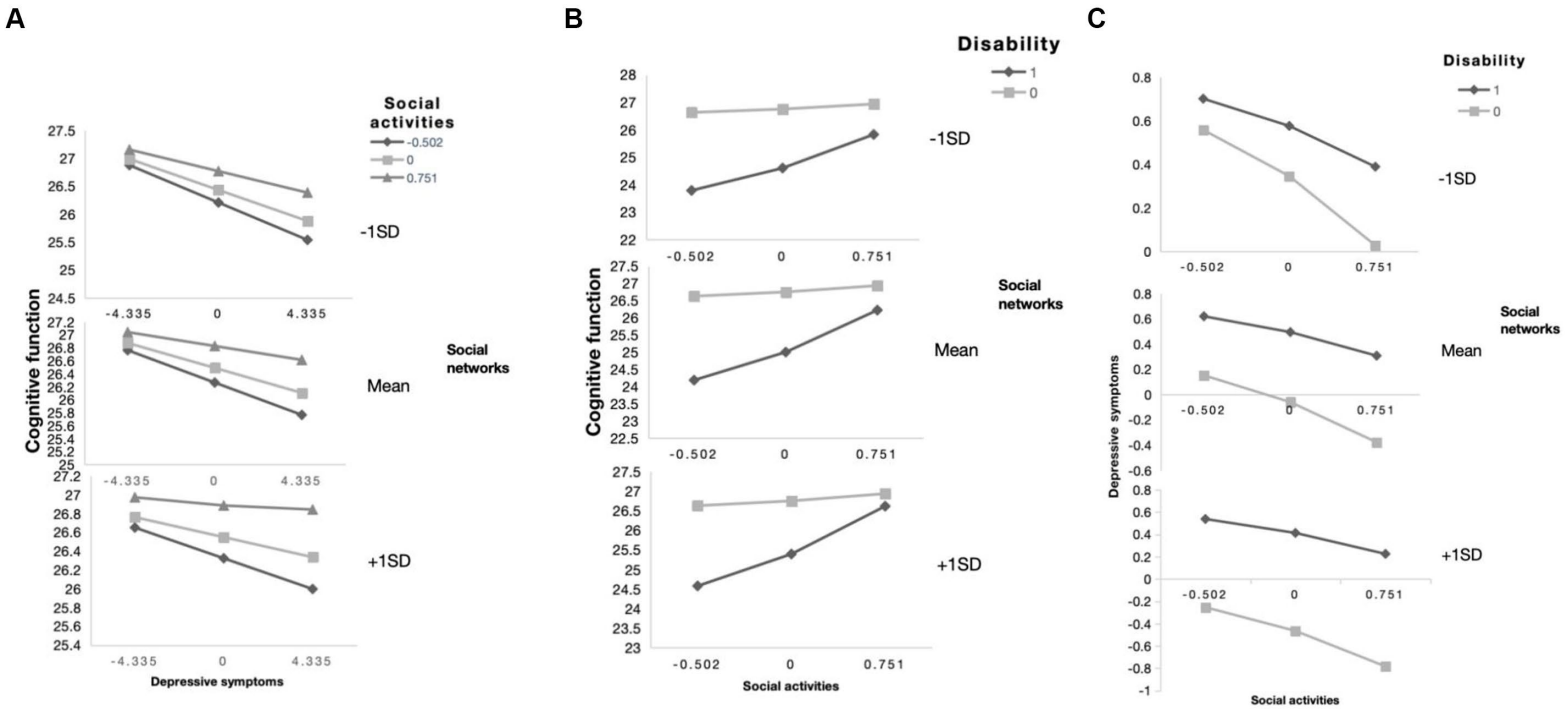
The simple plot of path **(A–C)** indicating the relationship between disability (0 = non-disabled,1 = disabled), depressive symptoms, and cognitive impairment among different levels of social activities and social networks group.

Table 5 shows that social relationships moderated the effect of disability on cognitive function to any extent, and as shown in Supplementary Table S6, social activities and social networks also moderated the impact of disability on cognitive function. In addition, the simple slope indicated that the cognitive function of the non-disabled older adults was always higher than that of the disabled older adults when social relationships were one standard deviation below (B = −4.548 *p* = 0.000) or one standard deviation above (B = −3.170 *p* = 0.000). Similarly, when social relationships were lower than one standard deviation (B = −0.190 *p* = 0.000) or higher than one standard deviation (B = −0.108 *p* = 0.000), cognitive function declined with the increase of depressive symptoms. In addition, according to Figure 5, cognitive function was worse when lower levels of social networks and social activities were associated with increased depressive symptoms. Figure 6 shows that more social activities were associated with fewer depressive symptoms. Cognitive function was worse when social activities and social networks were low and associated with disability.

**Table 5:** Conditional indirect effects of disability on social relationships.

Social relationships	B	SE	LLCI	ULCI
−1-SD	−0.064	0.028	−0.115	−0.015
Mean	−0.046	0.017	−0.083	−0.015
−1 + SD	−0.032	0.018	−0.074	−0.001

## Discussion

The results of this study show that depressive symptoms play a mediating role in the effect of disability on cognitive function. In addition, any degree of social relations will have a negative moderating role in the impact of disability on cognitive function. The higher the level of social relations, the stronger the negative moderating effect of disability on cognitive function. Social activities and social networks also played a negative moderating role in the impact of disability on cognitive function. Social activities and regulation moderated the direct and indirect effects of disability on cognitive function, while social support only moderated the immediate impact of disability on cognitive function.

This study found that the incidence of cognitive impairment in older adults was 18.6%, and the prevalence of cognitive impairment in women was higher than that in men, which was consistent with the results of previous studies (66). In addition, multiple influencing factors of cognitive impairment were explored, including age, gender, marital status, residence, education level, exercise, financial support, disability, sleep duration, frailty, comorbidity, and anxiety. These factors have also been validated in previous studies (67). Meanwhile, the prevalence of disability was 14.7% in older adults. Previous studies have shown that disabled older adults are more prone to depressive symptoms (68), possibly because disability can lead to a sense of physiological helplessness that increases the risk of depressed mood and cognitive deficits. However, no studies have investigated the mediating role of depressive symptoms in the effect of disability on cognitive impairment. To fill this research gap, older adults aged 65 years or older were selected because they are more likely to have disability, cognitive impairment, and depressive symptoms (69). Our findings support our hypothesis that depressive symptoms mediate the effect of disability on cognitive impairment. In addition, some studies have confirmed the moderating effect of social support on the relationship between disability and depressive symptoms (70, 71). Still, the moderating effect of social support on the relationship between disability and cognitive impairment has not been explored. To examine the moderating effect of social support more comprehensively, this study extended it to social relations, including social activities, social networks, and social support. This concept has been applied in previous studies (45, 72).

### Depressive symptoms mediate the link between disability and cognitive impairment

Based on the findings of the first model, depressive symptoms were found to mediate the relationship between disability and cognitive impairment. Specifically, higher levels of disability in older adults were associated with higher levels of cognitive impairment, suggesting a direct effect of disability on cognitive function. Depressive symptoms played an indirect role in the relationship between disability and cognitive impairment. A similar study conducted in China also found bidirectional associations among disability, cognitive impairment, and depressive symptoms (73). This mediating relationship can be divided into three paths: the first is the effect of disability on depressive symptoms. The results showed that the more disabled older adults were, the more severe the depressive symptoms were. This may be due to reduced contact with the outside world caused by restricted mobility, which induces feelings of helplessness and then induces depressive symptoms (74). In addition, other studies on activities of daily living (ADL) and depressive symptoms in older adults have also found that ADL impairment positively predicts depressive symptoms because older adults who need help may reduce self-confidence and self-worth and produce anxiety and depression (75, 76). Shimada et al. (77). in Japan found significant differences in daily activity function among older adults with different depressive symptoms. Together, these findings reveal a complex mechanism linking disability, cognitive impairment, and depressive symptoms.

The second path is the effect of depressive symptoms on cognitive impairment. The results of this study suggest that depressive symptoms in older adults can lead to cognitive impairment, which is because having depressive symptoms in older adults can reduce mental flexibility and their ability to consolidate and retrieve memories, thereby affecting the overall cognitive function of older adults (78). Previous studies have also confirmed that depression can seriously affect the occurrence of cognitive impairment (77, 79, 80). In addition, some studies have pointed out that the pathogenesis of cognitive impairment involves leukoaraiosis and non-pathological brain aging, which are related to the pathogenesis of depression, and there is an interaction between the two because they have similar pathological mechanisms (81, 82). Italian researchers Solfrizzi et al. surveyed 2,150 older adults. They found that there was a significant difference in the prevalence of cognitive frailty among older adults with different depressive symptoms, suggesting that cognitive impairment is related to the mental health of older adults.

The third pathway is the effect of disability on cognitive impairment. The results of this study suggest that disability can directly affect cognitive impairment and indirectly affect cognitive impairment through depressive symptoms. The direct impact of cognitive impairment is due to the increased risk of cognitive impairment in disabled older adults, such as limited physical function, decreased appetite, and malnutrition (79). The indirect effect of cognitive impairment is due to the decline of bodily function, activities, and social function in older adults, which leads to depression, which further reduces the frequency and time of social activities in older adults and ultimately increases the risk of cognitive impairment (82). Many studies have shown that cognitive impairment is closely related to activities of daily living in older adults, and the risk of disability is significantly increased in older adults with cognitive impairment (77, 83–86). In addition, a cross-sectional study involving 594 community-dwelling older adults in Italy showed that there was an interaction effect between cognitive impairment and disability in older adults, and older adults with cognitive frailty had a significantly higher level of disability than those without (83).

### The moderating role of social relations

For the second model of this study, social relationships were hypothesized as the moderating effect of the mediation model. The results suggest that social relations have a moderating impact on the three pathways of this mediation process. In other words, for disabled older adults with poor social relationships, having depression may lead to more significant cognitive impairment. Fan et al. (87) pointed out that family relationships significantly impact the cognitive function of older Chinese adults due to the influence of traditional Chinese culture. Harvard University professor Bond H (88), who has studied social relationships for 76 years, believes that good relationships can reduce disease incidence, improve negative emotions, and increase happiness. Previous studies have shown that Schroedl et al. (89), when studying the social participation ability of older adults, found that the impairment of the daily living ability of older adults can limit their social activities and thus reduce their social participation ability. In addition, studies have also found that the depressive state of older adults is affected by social participation (90), that is, social participation is related to depression. Most older adults believe that active social participation can prevent and relieve depression in older adults and help improve physical and mental health. In addition, structural equation modeling studies have validated the mediating effects of social interaction, depressive state, and lifestyle on the association between cognitive function and disability (91), suggesting that healthcare professionals should actively promote the social participation of disabled older people to improve their psychological status while providing daily care.

This study also found that social activities and social networks played a moderating role in the mediation model. The results showed that social activities moderated the direct effect of disability on cognitive impairment and the impact of depression on cognitive impairment. Social activities and disability negatively modulate the cognitive impairment pathway; in other words, older adults with fewer social activities have more severe cognitive impairment, similar to the results of Evans et al. (92). Carlson et al. (93) used experimental methods to find that participation in social activities such as voluntary service of particular intensity and requiring specific cognitive ability can delay the cognitive decline of older adults. In addition, social activities can not only delay the onset of Alzheimer’s disease in older adults but also help alleviate and improve mild cognitive impairment (94). In addition, the LANCET Commission on Dementia identified social isolation as one of the modifiable risk factors for dementia (95), and the results of a systematic review and meta-analysis of 51 longitudinal cohort studies on social isolation and cognition showed that aspects of social isolation were associated with cognitive function in later life (92).

Social activities and depression positively regulate the pathway of cognitive impairment. That is, the more social activities older adults have, the better their cognitive function. Relevant studies generally believe that older adults’ participation in volunteer and other social activities can mobilize their enthusiasm. While constantly meeting their social needs, they will realize their self-worth, and their demands for respect and recognition will be met accordingly. Finally, their cognitive ability will be improved (94). In addition, when older adults participate in social activities or make many friends, they obtain more social resources and higher social prestige to enhance the sense of social participation of older adults (96). Social infection theory believes that involvement in social activities can help older adults establish new social roles, improve their life satisfaction, and thus affect their mental health. It emphasizes that social participation prevents older adults from being isolated from society and can simultaneously adapt to changes in the social environment to reduce their social exclusion and loneliness. It has an impact on the mental health of older adults. Finally, the cognitive level of older adults was improved (97, 98).

Social networks moderated the indirect effect of disability on cognitive impairment in our study. Both pathways were positive regulation. That is, the more social networks, the lower the degree of depression and the better the cognitive function of older adults. The social network refers to the size of the contact population, the relationship with the subject, and the frequency of contact (99). Social support theory believes that social interaction can strengthen people’s social relationship networks and social resources to meet their development. Social participation is the primary way of social interaction for older adults, especially group communication social participation, which further strengthens the social network of older adults, especially the friend network, meets their psychological dependence needs and affects their mental health (100, 101). Studies have found that a rich social network positively affects the physical and mental health of older adults, and the more extensive the social network, the better the health of older adults and the fewer depressive symptoms (102). According to kinship, social networks can be divided into family and friend networks (103). In Western countries, scholars generally believe that friend networks play a greater role in supporting older adults and bringing more significant benefits to their physical and mental health (104, 105). However, in Chinese culture, the family network is a more important social network for older adults. Therefore, improving family support is more helpful to promote the physical and mental health of older adults and reduce loneliness and depression (106). Previous studies have shown that people with larger and more frequent social networks have higher cognitive scores. In contrast, people with less social networks are more likely to have mild cognitive impairment (107). The study by Rohr et al. (108) also showed that individuals with more extensive social network sizes had better cognitive function. In addition, it has been suggested that the effect of social networks on cognition may be because having a good social network (109) may be associated with a lower rate of depression and a larger brain and gray matter volume (110).

In conclusion, the results of this study suggest that social relationships must be optimized to prevent cognitive impairment in older adults. First of all, it is necessary to strengthen the interpersonal communication of older adults, not only supporting the visit of siblings and children but also strengthening the community communication activities to expand the social scope of older adults, which can not only promote the mental health of older adults but also improve their cognitive function. Second, the community should hold more activities and enrich the content of the activities, such as some activities to enhance the intelligence of older adults. Third, government departments should further improve the policies related to the activities of older adults in the community, such as carrying out some community activities, optimizing the allocation of resources in the community, etc., to improve the enthusiasm of older adults.

## Conclusion

This model provides a more visible picture of the factors that influence disabling cognitive impairment in older adults and offers an opportunity for healthcare professionals to detect, intervene, and monitor cognitive impairment early. In addition, the findings suggest that healthcare professionals should consider the interaction between social relationships and psychological effects in the causes of cognitive impairment to benefit older families, the healthcare system, and society at large.

## Limitations

There are some limitations of this study that are worth exploring. First, a 2018 cross-sectional study of the CLHLS database was used because the required data was missing for some years. Therefore, this study did not consider the chronological order of cognitive impairment, disability, depressive symptoms, and social relationships in older adults. It could not analyze the causal relationship between variables; only correlations were shown. In the future, longitudinal designs can be used to explore the causal relationship between disability and cognitive impairment and more accurate moderate-mediation relationships. Second, self-reported measures are prone to inaccuracies, and in-depth visits and behavioral observations should be conducted. Finally, the MMSE used in this study is only a screening tool and does not diagnose cognitive impairment. This measurement tool alone does not identify cognitive impairment at a particular moment.

## Data availability statement

All the data used in our study were obtained from a public database named Peking University Open Research Data (https://doi.org/10.18170/DVN/WBO7LK).

## Author contributions

FA: Conceptualization, Data curation, Investigation, Software, Visualization, Writing – original draft, Writing – review & editing. EL: Data curation, Writing – review & editing. AD: Writing – review & editing. HZ: Supervision, Writing – review & editing.
