# Supplementary material for: Association between disability and cognitive function in older Chinese people: a moderated mediation of social relationships and depressive symptoms
Source: Front Public Health. 2024 Apr 16;12:1354877. doi: 10.3389/fpubh.2024.1354877 (PMC11058663; doi:10.3389/fpubh.2024.1354877)
Supplement: Supplementary file 1 [file Data_Sheet_1.pdf]

Supplementary Material

- **Table S1 Description of study variables**

- **Table S2 The coding of social relationships**

- **Table S3 Testing the moderated mediating effect of disability on cognitive function by depression and social relationships**

- **Table S4 Testing the moderated mediating effect of disability on cognitive function by depression, social activities and social networks**

- **Table S5 Testing the moderated mediating effect of disability on cognitive function by depression and social support**

- **Table S6 Conditional indirect effects of disability on cognitive function at different levels of social activities and social networks**

Table S1 The Chinese version of the mini-mental state examination in the CLHLS

Domains	Questions	Scores
Orientation	What time of day is it right now (morning, afternoon, evening)?	1
	What is the month (Western or Chinese calendar) right now?	1
	What is the date (Chinese calendar day and month) of the mid-autumn festival?	1
	What is the season right now, spring, summer, fall, winter?	1
	What is the name of this district or town?	1
	Please name as many kinds of food as possible in 1 minute.	7
Registration	repeat table, apple and clothes	3
Attention and calculation	I will ask you to spend 3 dollars from 20 dollars, then you must spend 3 dollars from the number you arrived at and continue to spend 3 dollars until you are asked to stop.	5
	Asking the interviewee to copy a figure, in which all the sides and angles are correct	1
Recall	repeat the three words (in any order) that you heard a little while ago	3
Language	Naming pen and watch.	2
	Repeating the following sentence: "What you plant, what you will get."	1
	The individual is asked to take a paper using right hand, fold it in the middle using both hands, and place the paper on the floor.	3

Table S2. The coding of social relationships

Variable	Coding
**Social Relationships (score: 0-13)**	
**Social activities (score: 0-3)**	
Engagement in play cards/mah-jong	0=never; 1= almost every day /not daily, but once for a week/not weekly, but at least once for a month/not monthly, but
Engagement in organized activities	0=never; 1= almost every day /not daily, but once for a week/not weekly, but at least once for a month/not monthly, but
The number of having visiting experience	0=0; 1= more than 0
**Social networks (score: 0-4)**	
Marital status	0=widowed/divorced/never married; 1= married
Living arrangement	0=alone; 1=with household member /in an institution
Having sibling visiting	0=nobody; 1= more than 0
Having children visiting	0=nobody; 1= more than 0
**Social support (score: 0-6)**	
Having people to talk	0=nobody; 1= spouse/son/daughter/daughter in law/son in law/grandchildren/other relatives/friends/social workers/housekeeper
Having people to share thoughts	0=nobody; 1= spouse/son/daughter/daughter in law/son in law/grandchildren/other relatives/friends/social workers/housekeeper
Having people to ask for help	0=nobody; 1= spouse/son/daughter/daughter in law/son in law/grandchildren/other relatives/friends/social workers/housekeeper
Having people to be cared when having sick	0=nobody; 1=spouse/son/daughter/daughter in law/son in law/son and daughter/grandchildren/other relatives/friends and neighbors/social services/ live-in care giver
Financial support from son	0=0 yuan; 1= more than 0 yuan
Financial support from daughter	0=0 yuan; 1= more than 0 yuan

Table S3. Testing the moderated mediating effect of disability on cognitive function by depression and social relationships

	Depressive symptoms					Cognitive function				
	B	SE	*p*	LLCI	ULCI	B	SE	*p*	LLCI	ULCI
Disability (X)	0.551	0.152	0.000	0.252	0.849	-1.944	0.175	0.000	-2.287	-1.601
Depressive symptoms(M)	-	-	-	-	-	-0.091	0.015	0.000	-0.121	-0.061
Social relationships(W)	-0.174	0.035	0.000	-0.242	-0.106	0.118	0.040	0.000	0.040	0.197
X×W	0.150	0.086	0.080	-0.018	0.318	0.517	0.100	0.000	0.322	0.711
M×W	-	-	-	-	-	0.030	0.007	0.000	0.017	0.044

Table S4. Testing the moderated mediating effect of disability on cognitive function by depression, social activities and social networks

	Depressive symptoms					Cognitive function				
	B	SE	*p*	LLCI	ULCI	B	SE	*p*	LLCI	ULCI
Disability(X)	0.554	0.162	0.001	0.237	0.872	-1.750	0.183	0.000	-2.109	-1.392
Depressive symptoms(M)	-	-	-	-	-	-0.089	0.015	0.000	-0.119	-0.059
Social activities(W)	-0.424	0.071	0.000	-0.563	-0.285	0.245	0.082	0.003	0.084	0.406
Social networks(Z)	-0.384	0.053	0.000	-0.487	-0.280	0.000	0.060	0.999	-0.117	0.117
X×W	0.174	0.234	0.456	-0.284	0.633	1.381	0.267	0.000	0.859	1.904
X×Z	0.307	0.148	0.038	0.017	0.597	0.374	0.168	0.026	0.045	0.702
M×W	-	-	-	-	-	0.052	0.018	0.003	0.018	0.087
M×Z	-	-	-	-	-	0.038	0.011	0.001	0.016	0.059

Table S5. Testing the moderated mediating effect of disability on cognitive function by depression and social support

	Depressive symptoms					Cognitive function				
	B	SE	*p*	LLCI	ULCI	B	SE	*p*	LLCI	ULCI
Disability(X)	0.408	0.149	0.006	0.117	0.699	-2.216	0.167	0.000	-2.544	-1.889
Depressive symptoms(M)						-0.100	0.015	0.000	-0.130	-0.070
Social support(Y)	0.077	0.056	0.170	-0.033	0.187	0.181	0.063	0.004	0.058	0.305
X×Y	-0.079	0.143	0.579	-0.360	0.201	0.371	0.161	0.021	0.055	0.688
M×Y						0.019	0.012	0.127	-0.005	0.043

Table S6 Conditional indirect effects of disability on cognitive function at different levels of social activities and social networks

Social activities	Social networks	B	SE	LLCI	ULCI
-1-SD	-1-SD	-0.022	0.033	-0.089	0.041
-1-SD	Mean	-0.054	0.022	-0.102	-0.013
-1-SD	-1+SD	-0.060	0.028	-0.122	-0.014
Mean	-1-SD	-0.030	0.031	-0.096	0.027
Mean	Mean	-0.049	0.018	-0.089	-0.017
Mean	-1+SD	-0.043	0.021	-0.091	-0.008
-1+SD	-1-SD	-0.032	0.038	-0.120	0.033
-1+SD	Mean	-0.034	0.022	-0.087	0.000
-1+SD	-1+SD	-0.010	0.021	-0.056	0.029
